# Identification of putative phosphoproteins in wheat spikes induced by *Fusarium graminearum*

**DOI:** 10.1007/s00425-015-2441-y

**Published:** 2015-12-15

**Authors:** Lina Ding, Ruiying Yang, Guoxing Yang, Jun Cao, Peng Li, Yang Zhou

**Affiliations:** College of Life Sciences, Jiangsu University, Zhenjiang, 212013 China; Laboratory Middle School, Juancheng, 274600 Shandong China; College of Veterinary Medicine, Nanjing Agricultural University, Nanjing, 210095 China; Biotech Research Institute, Shanghai Academy of Agricultural Sciences, Shanghai, 201106 China

**Keywords:** *Fusarium* head blight, Phosphoprotein, Phosphoproteomics, Scab resistance, Wheat

## Abstract

**Phosphorylation and dephosphorylation events were initiated in wheat scab resistance. The putative FHB-responsive phosphoproteins are mainly involved in three functional groups and contain at least one tyrosine, serine, or threonine phosphorylation site.**

*Fusarium* head blight (FHB), caused by *Fusarium graminearum*, is a severe disease in wheat. Protein phosphorylation plays an important role in plant–pathogen interactions, however, a global analysis of protein phosphorylation in response to FHB infection remains to be explored. To study the effect of FHB on the phosphorylation state of wheat proteins, proteins extracted from spikes of a resistant wheat cultivar after 6 h of inoculation with *F. graminearum* or sterile H_2_O were separated by two-dimensional gel electrophoresis, and then the immunodetection of putative phosphoproteins was conducted by Western blotting using specific anti-phosphotyrosine antibody, anti-phosphothreonine antibody and anti-phosphoserine antibody. A total of 35 phosphorylated signals was detected and protein identities of 28 spots were determined. Functional categorization showed that the putative FHB-responsive phosphoproteins were mainly involved in defense/stress response, signal transduction, and metabolism. The phosphorylation status of proteins associated with signaling pathways mediated by salicylic acid, calcium ions, small GTPase, as well as with detoxification, reactive oxygen species scavenging, antimicrobial compound synthesis, and cell wall fortification was regulated in wheat spikes in response to *F. graminearum* infection. The present study reveals dynamics of wheat phosphoproteome in response to *F. graminearum* infection and suggests an important role of protein Ser/Thr/Tyr phosphorylation in fundamental mechanisms of wheat scab resistance.

**Electronic supplementary material:**

The online version of this article (doi:10.1007/s00425-015-2441-y) contains supplementary material, which is available to authorized users.

## Introduction

*Fusarium* head blight (FHB) or scab, caused by *Fusarium graminearum*, is a devastating disease in wheat (*Triticum aestivum* L.) and has been identified as a major factor limiting wheat production in many parts of world (Bai and Shannar 1994). Histological analysis showed that *F. graminearum* is a semi-biotroph. During the early stages of infection of detached barley leaves by *F. graminearum*, hyphal growth occurs without host cell necrosis and the fungus behaves like a biotroph. However, as infection progresses, infected spikes and detached leaf tissue become increasingly necrotic and bleached (Pritsch et al. 2000; Kang and Buchenauer 2000). During the infection of wheat spikes, *F. graminearum* produces cell-wall-degrading enzymes to facilitate penetration (Jaroszuk-Ściseł and Kurek 2012). In addition, the trichothecene mycotoxins produced by *F. graminearum* and *F. culmorum* (which are also called FHB) are known to inhibit protein synthesis and may have a role in pathogenesis, leading to a reduction in grain yield and quality (Boenisch and Schäfer 2011; Scherm et al. 2013). Although there is an effect of chemical control, breeding for FHB-resistant cultivars is still the best means to control this disease (Kollers et al. 2013; Lu et al. 2013; Niwa et al. 2014).

Wheat responds to *F. graminearum* infection by inducing various defense reactions, including morphological, physiological and biochemical effects and active defense reactions by the host. For example, significant differences in lignin monolignols composition, arabinoxylan (AX) substitutions, and pectin methylesterification were found between resistant and susceptible plants, suggesting that cell wall biochemical traits may relate to FHB resistance (Lionetti et al. 2015). Identification of host genes and proteins differentially expressed in response to FHB infection may help to illustrate cellular processes, activated or repressed during the early stage of FHB infection. Using large-scale genomic techniques, several classes of stress-related gene responses to *F. graninearum* infection have been discovered. These genes form a complex regulatory network involved in signal transduction, metabolism, transport, and defense response (Kong et al. 2005; Gottwald et al. 2012; Schweiger et al. 2013; Xiao et al. 2013). The transcripts of many defense response- and stress-related genes increased or are induced within 6–12 h after inoculation (hai) with *F. graminearum* in wheat spikes (Pritsch et al. 2000; Wang et al. 2005). Bernardo et al. (2007) reported that the up-regulation of defense-related genes occurred during the early stage (3–12 hai) of fungal stress as found when monitoring the expression patterns of transcriptomes from wheat spikes during a period of 72 hai with *F. graminearum*. Mostly, the transcripts’ accumulation rates were higher in the FHB-resistant as compared to the susceptible genotype (Muhovski et al. 2012). Transgenic wheat expressing defense response genes, such as RsAFP2, TaLTP5, and lactoferrin, which inhibit fungal infection in a variety of ways, can enhance resistance to FHB under greenhouse and/or field conditions (Han et al. 2012; Zhu et al. 2012).

Proteomic approaches have been widely used to study plant–pathogen interactions. Comparative proteome analysis has enabled direct isolation and identification of proteins associated with resistance to FHB (Zhou et al. 2005; Ding et al. 2011; Zhang et al. 2013). Zhang et al. (2013) compared protein profiles between near-isogenic lines (NILs) contrasting in alleles of *Fhb1*, a major FHB resistance gene in wheat, and found that wheat proteins for defending fungal penetration, photosynthesis, energy metabolism, and detoxification were differentially expressed in the Fhb1(+) NIL. By a combined proteomic and transcriptomic approach, the FHB resistance was found to be associated with coordinated and ordered activation signaling events involving Ca^2+^, salicylic acid (SA), and jasmonic acid (JA)/ethylene (ET) pathways during the first 24 hai (Ding et al. 2011).

Proteomics not only monitor the steady state level of proteins but also co- and post-translational modifications of proteins. Regarding the possible modifications, phosphorylation of proteins has gained the most attention since many proteins rely on phosphorylation and dephosphorylation for activation/inactivation and transmitting signals within cellular pathways and networks (Zhao et al. 2013; Yasuda et al. 2014). In plants, early events following pathogen attack generally include the phosphorylation of certain cytosolic proteins, which play an important role in defense response through activation of downstream genes (Ishihama et al. 2011; Wang et al. 2014a, b; Kang et al. 2015). Phosphorylation and dephosphorylation in plant defense is also regulated so that it is coordinated with other activities of the host cells (Xing et al. 1996). Owing to the importance of protein phosphorylation in regulating cellular processes, a major goal of current proteomic research is to identify putative phosphoproteins in organisms and understanding their functions.

Differential proteomic analysis provides valuable information about protein expression in wheat spikes responding to FHB infection, however, a global analysis of protein phosphorylation in response to FHB infection has not yet been studied in detail. In the present study, we detected and identified putative phosphoproteins in FHB-resistant wheat cultivar Yangmai 18 under infected and uninfected conditions. Furthermore, the changes of protein phosphorylation responding to *F. graminearum* infection have been investigated and discussed. Phosphoproteomic analysis of dynamic phosphorylation events and identification of putative phosphoproteins may provide novel understanding about wheat defense mechanisms to FHB infection.

## Materials and methods

### Plant materials and protein extraction

Wheat spikes from resistant cultivar (*Triticum aestivum* L. cv. Yangmai 18) were treated and collected as described in Ding et al. (2011) and stored at −80 °C. Protein extraction was conducted according to Damerval et al. (1986). Briefly, modified as following, frozen spikes were ground in liquid nitrogen and proteins were precipitated at −20 °C with 10 % (w/v) trichloroacetic acid (TCA) in acetone containing 0.07 % (w/v) DTT for 1 h. The mixture was centrifuged at 15,000*g* at 4 °C for 30 min, and the precipitate was washed with ice-cold acetone containing 0.07 % (w/v) DTT to remove pigments and lipids until the pellet become colorless. The pellets were dried by vacuum centrifugation, and resuspended in extraction buffer containing 8 M urea, 4 % (w/v) CHAPS, 20 mM DTT, 0.2 % (v/v) carrier ampholyte (pH 3.0–10.0), protease inhibitor cocktails (1 µL per 30 mg plant tissues, Sigma) and phosphatase inhibitor cocktails (10 µL per 1 ml of extraction buffer, Sigma) at room temperature for 30 min, and sonicated five times for 30 s each on ice. After extraction, the solution was centrifuged at 40,000*g* for 30 min. The supernatant was stored at −80 °C. Protein concentration of the extracts was quantified with bovine serum albumin as standard using the Bradford method (Bradford 1976).

### Two-dimensional gel electrophoresis (2-DE)

For total protein separation, the immobilized pH gradient (IPG) strips (pH 3–10, linear, 7 cm, Bio-Rad) were rehydrated passively for 13 h. The voltage settings for isoelectric focusing (IEF) in the Protean system (Bio-Rad) were 1 h at 250 V, 1 h at 500 V, 1 h at 2000 V, 2 h at 5000 V, and then hold at 5000 V until a total voltage of 25,000 Vh was reached. After IEF and equilibration, the second dimensional SDS-PAGE gels of 12.5 % were run at 3 W/gel for 45 min and 12 W/gel for 1.5 h using Multiphor system (Amersham Biosciences). The gels were visualized by colloidal Coomassie brilliant blue staining.

### Western blotting

For each sample, duplicate 2-DE gels were run under the same conditions. One gel was subjected to colloidal Coomassie staining to visualize the protein spots and analyze the spots using mass spectrometry (MS). The other gel was transferred onto polyvinylidene fluoride (PVDF) membrane (Millipore) at 4 °C and 30 V for overnight. After electrotransfer step, all transferred protein spots on PVDF membranes were stained temporarily with Ponceau S solution and then scanned. These Ponceau S-stained images served as reference gel images to match the putative phosphoprotein spots and Coomassie-stained protein spots.

The transferred PVDF membranes were blocked for 1 h at 37 °C with blocking solution (3 % BSA in TBST, 0.05 % Tween-20), and incubated with anti-phosphotyrosine (p-Tyr) antibody (phosphotyrosine detection kit, Calbiochem, code no. #525322), anti-phosphothreonine (p-Thr) antibody (phosphothreonine detection kit, Calbiochem, code no. #525288) and anti-phosphoserine (p-Ser) antibody (phosphoserine detection kit, Calbiochem, code no. #525282), respectively, for 2 h at room temperature at a dilution of 1:5000 in TBST. After washing four times in TBST, the membranes were incubated with a horseradish peroxidase conjugated secondary antibody (Promega, Catalog #W4011, 1:2500 dilution) for 2 h at room temperature. The PVDF membranes was extensively washed four times in TBST, then the putative phosphoprotein spots were detected with an enhanced chemiluminescence kit (SuperSignal™ West Pico substrate; Pierce Biotechnology) for 5 min, then scanned. Image analysis software PDQuest (Bio-Rad) was used to match protein spots correctly between Coomassie-stained spots in gels and Ponceau S-stained protein spots in PVDF membrane or between Ponceau S-stained protein spots and putative phosphoprotein spots in the same PVDF membranes. Since no matrix-assisted laser desorption ionization time-of-flight (MALDI-TOF)-MS spectra could be obtained from proteins blotted onto PVDF membranes, protein spot identities were assigned by matching the chemiluminescence images with Coommassie-stained gels run in parallel. All experiments with triplicate were done twice as independent biological replicates.

### MALDI-TOF-MS analysis and protein identification

All the steps from in-gel digestion of proteins to MALDI-TOF-MS analysis were as described in Ding et al. (2011). Calibration was carried out using a standard peptide mixture. The mass spectra (MS) data were collected from mono isotopic peaks falling in the m/z range of 750–4000 Da with S/N ratio over 10. Peaks resulting from autolysis of trypsin and from commonly occurring keratin contamination were excluded from the mass list. Protein identification of the peptide mass fingerprint (PMF) data was performed using the Mascot search engine (Matrix Science, London, UK, v2.4). The following parameters were met: a monoisotopic mass accuracy of ±1 Da; up to one missed cleavage; a fixed modification of carbamidomethyl (Cys) and variable modifications of oxidation (Met). Raw MS data files were converted to a DTA files, which were used to query the NCBI non-redundant database, limited to *Viridiplantae*. Proteins were considered correctly identified if returns contained two or more peptides with a significant score as defined.

### Bioinformatics analysis of the identified putative phosphoproteins

To address the functional characteristics of the identified proteins, protein functions were assigned using the protein function database Pfam (http://www.sanger.ac.uk/Software/Pfam/) or Inter-Pro (http://www.ebi.ac.uk/interpro/) (Apweiler et al. 2001; Bateman et al. 2002). Identified proteins were categorized according to their biological functions as described by Ding et al. (2011). The subcellular locations of the unique proteins identified in this study were predicted using the publicly available program, WolfPsort (http://wolfpsort.org) (Wu et al. 2013).

For evaluation of putative phosphoproteins that were identified to be phosphorylated, predictions of phosphorylation sites of identified proteins were conducted using NetPhos prediction server 2.0 (http://www.cbs.dtu.dk/services/NetPhos/) and ScanPROSITE tool (http://www.expasy.org/tools/scanprosite). Further database searches with PhosphoBase (http://cbs.dtu.dk/databases/PhosphoBase), BRENDA (http://brenda.uniko.de), PubMed (http://www.ncbi.nlm.nih.gov/PubMed/) were carried out.

## Results

### Comparison of phosphoproteomic patterns with and without *F. graminearum* infection

**Fig. 1:**
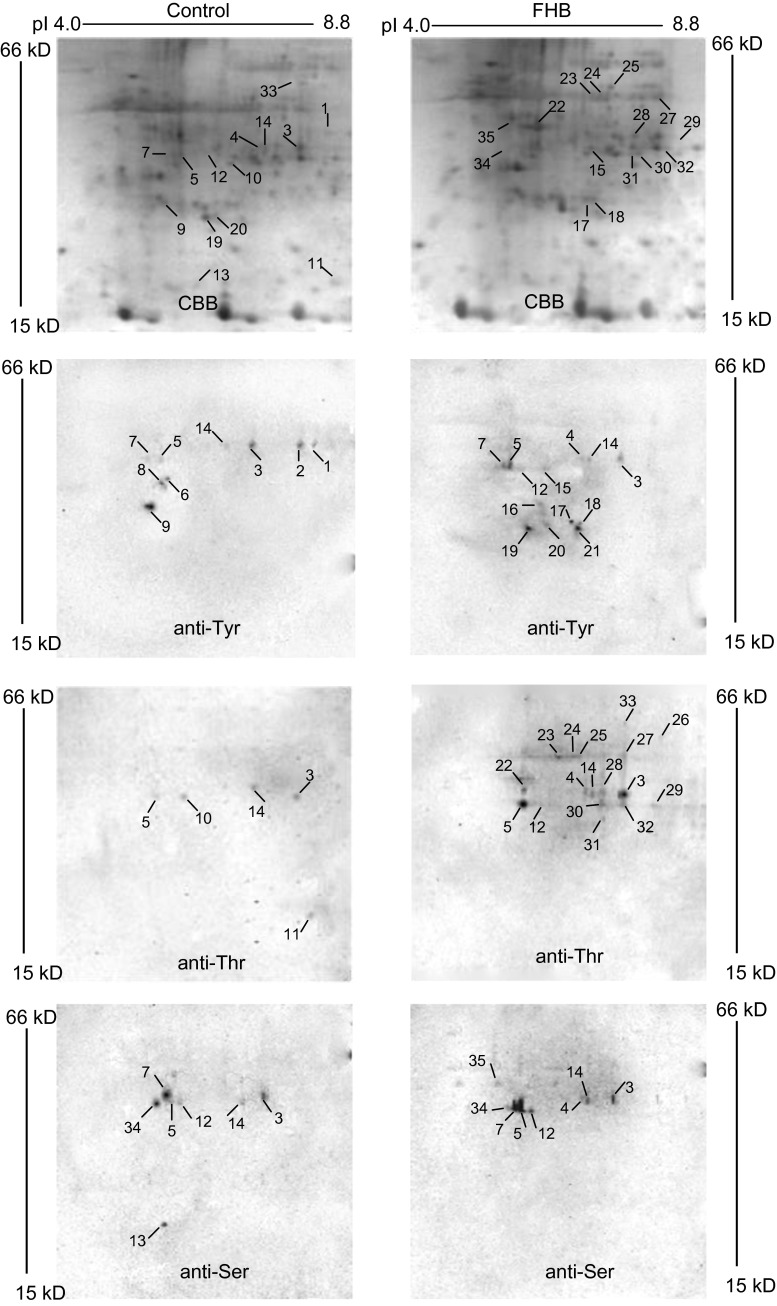
2-DE images visualized by Coomassie blue staining and immunostaining. **a**, **b** Coomassie blue-stained protein profiles of wheat spikes with water treatment and 6 hai after infection. **c**, **d** Phosphotyrosine 2-DE images of wheat spikes with water treatment and 6 hai after infection. **e**, **f** Phosphothreonine 2-DE images of wheat spikes with water treatment and 6 hai after infection. **g**, **h** Phosphoserine 2-DE images of wheat spikes with water treatment and 6 hai after infection. Representative figure from three technical and two biological replicates

One hundred micrograms of each protein extract of wheat spikes was separated by 2-DE and transferred to PVDF membranes, then phosphorylated signals were detected by immunostaining using anti-pTyr antibody, anti-pThr antibody and anti-pSer antibody respectively (Fig. 1). Parallel gels were stained with colloidal Coomassie blue and analyzed using PDQuest software. On average, 200 protein spots were detected in pH 3–10 2-DE images of proteins of wheat spikes with and without *F. graminearum* infection, and the protein patterns between the treatment and control were similar (Fig. 1a, b). In the phosphoproteomic patterns with and without *F. graminearum* infection, a total of 35 phosphorylated signals were detected (27 and 15 phosphorylated signals, respectively). Comparing the expression levels between the samples with and without *F. graminearum* infection, no higher difference for these proteins than 1.5-fold was found (Fig. S1). In immunostaining 2-DE images of proteins without infection, 9, 5 and 7 phosphorylated signals were discovered using anti-pTyr antibody, anti-pThr antibody, and anti-pSer antibody, respectively; spots 3, 5, 14 of them were all detected by three types of antibodies (Fig. 1c, e, g). Parallel studies were done with FHB infection, 13, 17 and 8 phosphorylated signals were detected, respectively, and spots 3, 4, 5, 12, 14 of them were all detected by three types of antibodies (Fig. 1d, f, h).

**Fig. 2:**
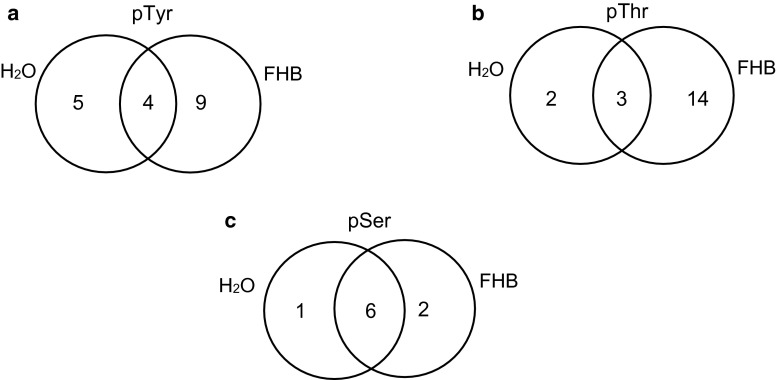
Venn diagram of the differentially expressed putative phosphoproteins in Yangmai 18 with water treatment and 12 hai after *F. graminearum* infection. **a** Phosphotyrosine proteins. **b** Phosphothreonine proteins. **c** Phosphoserine proteins

Using anti-pTyr antibody, four spots were found with and without *F. graminearum* infection (spots 3, 5, 7, and 14). Five spots were detected without *F. graminearum* infection (spots 1, 2, 6, 8 and 9); however, nine spots were detected with *F. graminearum* infection (spots 4, 12, 15, 16, 17, 18, 19, 20 and 21) (Fig. 2a). The intensity of the phosphorylated signal for spot 3 decreased 1.5-fold with FHB infection, however, spots 5 and 7 increased 1.7- and 3.0-fold in signal intensity (Fig. 1c, d). Using immunostaining, anti-pThr antibody discovered nineteen phosphorylated signals with and without *F. graminearum* infection, of which three spots were detected in both treatments (spots 3, 5 and 14), two (spots 10 and 11) and fourteen (spots 4, 12, 22, 23, 24, 25, 26, 27, 28, 29, 30, 31, 32 and 33) phosphorylated signals were especially detected with and without *F. graminearum* infection, respectively (Fig. 2b). The signal intensities of spots 3 and 14 increased 1.9- and 4.2-fold, respectively, with FHB infection (Fig. 1e, f). Western blotting analysis using anti-pSer antibody detected nine phosphorylated signals, of which three spots were special phosphorylated signals (spots 4, 13 and 35), and eight spots emerged in both patterns (spots 3, 5, 7, 12, 14 and 34) (Fig. 2c). It is noticeable that the signal intensity of spot 14 increased 2.5-fold with FHB infection (Fig. 1g, h). Some phosphorylated signals showed significant changes due to FHB infection. These results indicated that the phosphorylation status or phosphorylation level of wheat spike proteins was regulated by phosphorylation and/or dephosphorylation, and that protein phosphorylation is dynamic during wheat defense reaction responding to *F. graminearum* infection.

### Identification of putative phosphorylated proteins by MALDI-TOF MS

A total of thirty-five phosphorylated signals was found by immunostaining, but six spots (spots 2, 6, 8, 16, 21 and 26) of them were obviously not detectable by colloidal Coomassie staining of 2-DE gels (Fig. 1a, b). This is because the sensitivity of immunostaining is higher than that of colloidal Coomassie staining, and antibodies can detect as little as a few fmol of an epitope (Yan et al. 1998). Thus, only 29 spots visualized by Western blot analysis were excised from 2D gels, subject to in-gel digestion and analyzed by MALDI-TOF MS to obtain peptide mass fingerprint (PMF) data.

For identification of a putative phosphorylated protein spot, we did not conduct the possible phosphorylation modification on tyrosine, threonine or/and serine residues in database searches. Database searches for phosphorylated protein identification using search engines Profound, Mascot, or MS-Fit, etc., showed that once “possible phosphorylation modification on tyrosine, threonine or/and serine residues” was chosen, most residues of tyrosine, threonine or serine existed in sequence of a candidate protein will be predicted to be phosphorylated. In fact, it is not the case in plant organism. In addition, we compared the influence of “phosphorylation modification” that was chosen and “phosphorylation modification” that was not chosen for phosphorylated protein identification by combining the MS fingerprinting data and MS/MS data (unpublished data). The results showed that the latter was prior to be considered. Therefore, identification of phosphorylated protein spots should be conducted with caution. Besides spot 29 which did not give a positive identification, proteins matched with 28 spots were identified. Table 1 lists the spot number, accession number according to GeneBank, functional description based on Gene Ontology (GO) and information reported in literature, Mascot protein scores, number of matched peptides, percentage of sequence coverage, theoretical and experimental Mr and p*I*, subcellular location and phosphorylated signal detected by each specific antibody.

**Table 1:** Phosphoproteins identified by PMF query

Spot no.	Exp. MW/p*I* ^a^	The. MW/p*I* ^b^	Coverage % (PM)^c^	Accession no.	Protein name	Possible function	Score	Subcellular location	Phosphorylated signal^d^		
									Tyr	Thr	Ser
1	42.7/8.4	42.7/8.1	34 (11)	BAB89081	dnaJ-like protein	Stress response	122	Nucleus	_	nd	nd
3	40.3/8.2	35.0/8.1	50 (13)	AAY57588	RING-finger E3 ubiquitin ligase	Signal transduction	215	Nucleus	_ +	_ +	_ +
4	40.0/7.5	39.0/6.3	50 (14)	CAA64683	osr40c1	Defense response	218	Cytoplasm	+	+	+
5	37.5/6.2	37.9/6.7	37 (10)	BAD61512	Putative auxin-induced protein	Signal transduction	118	Chloroplast	_ +	_ +	_ +
7	37.5/6.1	41.4/6.2	44 (19)	CAA77237	Reversibly glycosylated polypeptide	Cell wall formation, defense response	189	Cytoplasm	_ +	nd	_ +
9	27.7/6.0	20.5/6.8	26 (3)	AAM20041	Putative ADP-ribosylation factor	Signal transduction	62	Cytoplasm	_	nd	nd
10	35.2/6.7	41.4/7.0	58 (6)	AAD19957	Thiosulfate sulfurtransferase	Metabolism	110	Chloroplast	nd	_	nd
11	23.2/8.5	26.2/7.8	24 (3)	AAQ06276	Uncharacterized protein	Unknown	67	Chloroplast	nd	_	nd
12	37.0/6.4	35.7/6.6	26 (4)	CAD40878	NAD(P)-linked oxidoreductase	Stress response	68	Cytoplasm	+	+	_ +
13	24.9/7.2	30.1/8.1	43 (5)	AAO20076	Putative phosphatidylinositol/phosphatidylcholine transfer protein	Transport	72	Mitochondrion	nd	nd	_
14	39.6/7.6	42.1/7.0	32 (14)	BAD15446	Receptor protein kinase PERK1-like protein	Signal transduction	134	Cytoplasm	_ +	_ +	_ +
15	37.3/7.1	36.5/6.7	27 (10)	AAX19515	Serine/threonine protein kinase	Signal transduction	122	Cytoplasm	+	nd	nd
17	27.8/7.0	26.6/8.1	51 (10)	AAC96317	Heat shock protein HSP26	Stress response	156	Chloroplast	+	nd	nd
18	27.5/7.1	31.7/7.1	78 (11)	BAD68853	Hypothetical protein	Unknown	145	Cytoplasm	+	nd	nd
19	25.2/6.4	30.5/6.7	43 (16)	AAP44649	Putative ABC transporter	Transport	201	Chloroplast	+	nd	nd
20	25.6/6.7	29.2/7.3	25 (4)	BAD81942	Unknown protein	Unknown	65	Chloroplast	+	nd	nd
22	42.4/6.2	35.9/6.8	46 (18)	BAC99738	Putative cinnamoyl-CoA reductase	Lignin biosynthesis, defense response	225	Chloroplast	nd	+	nd
23	54.5/7.1	53.0/6.3	41 (18)	AAQ64632	Glutathione reductase	Detoxification, defense response	246	Cytoplasm	nd	+	nd
24	54.4/7.2	53.2/6.3	43 (11)	AAU44253	Putative trehalose-6-phosphate synthase	Cell wall biogenesis, defense response	170	Nucleus	nd	+	nd
25	54.8/7.3	49.8/7.1	46 (16)	CAA51931	Phosphoglycerate kinase	Metabolism	228	Chloroplast	nd	+	nd
27	52.1/8.4	48.2/8.5	24 (8)	BAC79536	Putative CBL-interacting protein kinase	Signal transduction	84	Chloroplast	nd	+	nd
28	40.4/7.7	33.4/7.7	37 (6)	AAW67000	Isochorismate synthase protein	Defense response, SA biosynthesis	126	Chloroplast	nd	+	nd
30	37.4/7.7	38.5/7.3	47 (18)	BAC83804	r40g2 protein	Metabolism	128	Cytoplasm	nd	+	nd
31	36.0/7.5	37.6/7.1	30 (4)	AAW52722	Peroxidase 8	ROS scavenging, defense response	85	Chloroplast	nd	+	nd
32	37.4/8.1	34.7/8.4	40 (12)	AAR01635	Cellular retinaldehyde-binding protein	Metabolism	136	Nucleus	nd	+	nd
33	60.8/8.2	57.5/9.0	23 (8)	AAR11387	Cytochrome P450	Defense response	95	Chloroplast	nd	+	nd
34	37.3/5.9	35.8/5.5	38 (8)	BAD05829	Zinc finger (C3HC4-type RING finger) protein -like	Stress response	119	Plasma membrane	nd	nd	_ +
35	45.6/5.8	46.6/7.0	37 (10)	BAD08982	Putative RNA recognition motif (RRM)-containing protein	Transcription	132	Nucleus	nd	nd	+

^a^Experimental relative molecular mass (kDa)/isoelectric point

^b^Theoretical relative molecular mass (kDa)/isoelectric point

^c^Sequence coverage by peptide mass fingerprinting using MALDI-TOF MS. PM, number of peptides matched

^d^Phosphorylated signal detected with anti-phosphotyrosine antibody, anti-phosphothreonine antibody and anti-phosphoserine antibody. − represents wheat spikes treated with H_2_O; + represents wheat spikes treated with *F. graminearum*; *nd* represents no detection

### Functional classification and analysis of the putative phosphoproteins

**Fig. 3:**
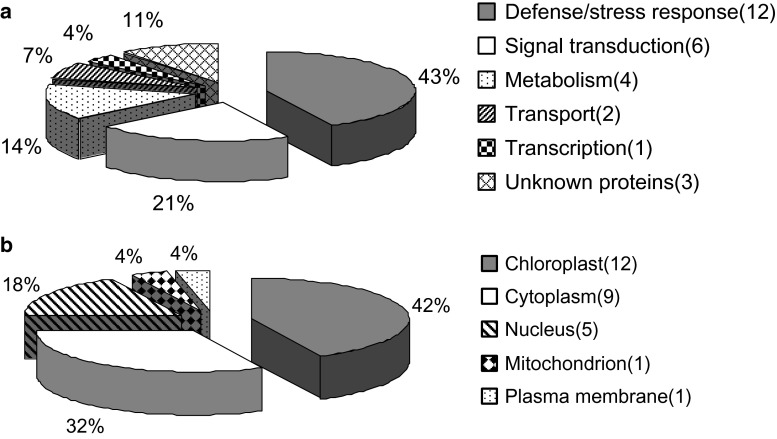
Distribution of functional categorization (**a**) and subcellular location of the identified putative phosphoproteins (**b**). The number of proteins in each category is indicated in *parentheses*

The 28 identified proteins were mainly classified into 6 categories based on their functions, including defense/stress response (1), signal transduction (2), metabolism (3), transport (4), transcription (5), and other proteins with unknown functions (6) (Fig. 3a). An impressive 64 % of these identified proteins was implicated in the first two functional groups, indicating that these processes were greatly regulated by protein phosphorylation or dephosphorylation events, and of functional importance in FHB resistance. Bioinformatic analysis by WolfPsort revealed that the majority of identified proteins were located in the chloroplast and cytoplasm (Fig. 3b).

The largest group of proteins discovered using phospho-specific antibodies in the absence or presence of *F. graminearum* was attributed to the proteins involved in defense/stress response. These include spots 1, 4, 7, 12, 17, 22, 23, 24, 28, 31, 33 and 34. Spot 4, showing phosphorylated signal after FHB infection, was identified as *osr40c1* (Fig. 1). Moons et al. (1997) reported that the expression of *osr40c1*, encoding an abscisic acid-responsive protein from rice, is rapidly enhanced by salt stress and decreased as wilting-induced changes. But plant growth regulators that trigger the wounding and pathogen defense reactions such as SA, ethylene, and JA, provoked a decrease in *osr40c1* transcript levels. Spots 1, 17 and 34 were identified as dnaJ-like protein, heat shock protein HSP26 and zinc finger (C3HC4-type RING finger) protein–like, respectively. These proteins are linked to stress response in plants and play important roles in various physiological processes (Cho et al. 2012; Yuan et al. 2013). It has been reported that some HSPs are involved in wheat resistance reaction against *F. graminearum* attack (Wang et al. 2005; Schweiger et al. 2013), and Lund et al. (2001) reported the first instance where a plant sHSP has been shown to be phosphorylated in vivo. Spots 7 and 24 were identified, respectively, as reversibly glycosylated polypeptide and putative trehalose-6-phosphate synthase, which were thought to be involved in cell wall formation. Phospho-specific antibodies also detected protein responses to oxidative stress, such as NAD(P)-linked oxidoreductase (spot 12), glutathione reductase (spot 23) and peroxidase 8 (spot 31), which exhibited a phosphorylated signal after FHB infection. These enzymes provide the plant cell with a highly efficient machinery for scavenging ROS and tightly control the equilibrium of the antioxidant system in plants. In addition, three defense response putative phosphoproteins related to secondary metabolism including putative cinnamoyl-CoA reductase (spot 33), isochorismate synthase (spot 22) and cytochrome P450 (spot 28), which are needed for lignin, SA and phytoalexin biosynthesis, respectively, produced a phosphorylated signal after FHB infection (Fig. 1). It is suggested that these secondary metabolic substances function in diverse physiological processes including disease resistance and stress responses (Kong et al. 2005).

The second largest group of identified proteins were involved in signal transduction, including RING-finger E3 ubiquitin ligase (spot 3), putative auxin-induced protein (spot 5), putative ADP-ribosylation factor (ARF, spot 9), receptor protein kinase PERK1-like protein (spot 14), serine/threonine protein kinase (STPK, spot 15) and putative CBL-interacting protein kinase (CIPK, spot 27). A protein homolog of spot 3 in wheat had a putative phosphorylation site at the C terminus, and was responsive to cold and/or dehydration (Guerra et al. 2012). ARF, whose phosphorylated signal disappeared responding to FHB infection (Fig. 1c, d), are small GTP-binding proteins that regulate a wide variety of cell functions in wheat (Pu et al. 2014). Receptor protein kinase PERK1-like protein, STPK and CIPK are all subclasses of the protein kinase superfamily that may function in their controlled reversible phosphorylation, and may further influence many aspects of cellular processes. The phosphorylated signal intensity of receptor protein kinase PERK1-like protein increased with infection (Fig. 1c–h). CIPK, showing phosphorylated signal in response to FHB infection (Fig. 1e, f), functions in a Ca^2+^-related pathway and responds strongly to both abiotic and biotic environmental stimuli through phosphorylation or dephosphorylation downstream target proteins at specific residues (Deng et al. 2013; Yu et al. 2014).

Putative phosphoproteins involved in metabolism were the third largest group (18 %) whereas the other biological processes were represented at a much lower scale. Thiosulfate sulfurtransferase matched to spot 10, and was related to resistance to powdery mildew in wheat (Niu et al. 2002). Spot 13 was matched to putative phosphatidylinositol/phosphatidylcholine transfer protein (PITP), whose phosphorylated signal disappeared after FHB infection (Fig. 1g, h). It was reported that this protein linked to stress response and developmental regulation in higher plants (Phillips et al. 2006). Spot 19, showing phosphorylated signals in response to FHB infection (Fig. 1c, d), was matched to a putative ABC transporter, which was considered to widely participate in the plant defense reaction under pathogen attack (Kang et al. 2011).

Spots 25, 30, 32 and 35, all showing phosphorylated signals with FHB infection, were matched to phosphoglycerate kinase, r40g2, cellular retinaldehyde-binding protein and putative RNA recognition motif (RRM)-containing protein, respectively. These proteins have not been reported for their association with plant defense reaction and modification of phosphorylation. The proteins identified for spots 11, 18 and 20 were matched to hypothetical proteins or unknown ones, which were categorized into an unclear function group.

### Phosphorylation sites of identified putative phosphoproteins

The probability for each of the identified proteins to be phosphorylated was evaluated using NetPhos and ScanPROSITE. NetPhos analysis predicted that all the identified proteins contain at least one tyrosine, serine or/and threonine phosphorylation site (data not shown). In order to determine whether any putative phosphorylation motif occurs in identified putative phosphoproteins, ScanPROSITE analysis was carried out, and predicted that 23 proteins contain the known kinase phosphorylation motifs.

## Discussion

In organisms, some proteins exist in phosphorylated form and non-phosphorylated form. Phosphorylation rates and phosphorylation abundances are very low (1–2 % of the entire protein amount is present in a phosphorylated form), especially in signaling pathways. While some residues are always quantitatively phosphorylated, others may only be transiently phosphorylated up to 0.5 % (Schlessinger 1993). Another report described that the amount of phosphorylated form of a protein is about one-tenth of the total amount of the same protein, and the modification of protein phosphorylation only happened on one or several distinct sites of the protein sequence (Wu and MacCoss 2002). Thus, only a small fraction of the population of proteins of interest is phosphorylated at a particular site.

The combination of immobilized metal affinity chromatography (IMAC) and mass spectrometry is a widely used technique for enrichment and sequencing of phosphopeptides (Rosenqvist et al. 2011; Zhou et al. 2011). In mass spectrometry analysis, the signal of the phosphopeptide is easily inhibited by the non-phosphorylated peptide. Therefore, effective enrichment and sequencing of phosphopeptide is highly important to ensure a successful study of phosphorylation-mediated protein regulation.

For phosphoproteomics studies, the immunodetection of putative phosphoproteins, following their electrophoretic separation, by Western blotting using antibodies against the phosphoamino acids, can be used to analyze proteins phosphorylated on tyrosine, serine or threonine residues (Bergström Lind et al. 2008; Lind et al. 2012; van der Mijn et al. 2015). This is one of the most sensitive techniques for detecting specific phosphorylation sites on tyrosine, serine or/and threonine residues. But these studies mainly focused on phosphoproteomics of humans, animals or bacteria. To our knowledge, there were only few reports of phosphoproteomics in plants, especially in wheat. Phosphoprotein analysis using phosphoproteomics techniques provides an insight into the molecular function of some phosphorylated proteins in wheat spikes infected by *F. graminearum*. Wheat responds to *F. graminearum* infection with alterations in the phosphorylation status of particular proteins. The identification of such particular proteins or signaling molecules displaying altered phosphorylation status after infection is important to elucidate the expression network associated to wheat resistance against FHB infection.

In the present study, only a small quantity of putative phosphoproteins was detected, possibly because the antibodies against p-Tyr, p-Ser and p-Thr residues, which were used in this approach, could not recognize all proteins that harbored phosphates on these residues or could not detect certain phosphoproteins due to steric hindrance of the recognition site (Kaufmann et al. 2001). Although the number might not be extraordinary, our annotation results are quite informative and illustrative. Most of the putative phosphoproteins, either with or without *F. graminearum* infection, were involved in disease/stress response, signal transduction, and metabolism. Thus, these processes may be important targets of the phosphorylation cascades in wheat responding to *F. graminearum* infection. The results are consistent with our previous studies about wheat proteins associated with resistance to FHB (Ding et al. 2011). The present study is distinguished from the previous study by the identification of a protein involved in SA synthesis (spot 28) and of proteins related to transport (spots 13 and 19). The lack of JA/ET pathway proteins identified in this study can most likely be attributed to the fact that we used a 6-h time interval between inoculation and sampling, while the previous study used a 12-h time interval. Hence, activation of the SA pathway may be a relatively early defense event and the activation of the JA/ET pathway a relatively late defense event, which suggests that in scab-resistant cultivars, the actions of SA and JA/ET pathways may better coordinated to diminish their potential antagonistic interactions.

Appropriate activation of early defense signaling events leads to disease resistance, which is implemented by cellular activities such as synthesis of phytoalexins, detoxification enzymes, and cell wall modifications (Ding et al. 2011). In the phosphoproteomics analyses, proteins related to these activities were detected. For example, putative cinnamoyl-CoA reductase (spot 33) and cytochrome P450 (spot 28), involved in lignin and phytoalexin biosynthesis, and glutathione reductase (spot 23) with the function of ROS scavenging. Since many phytoalexins are toxic to pathogens, and lignin is closely associated with the protection of wheat against pathogen infection, these processes identified in our study may provide a good strategy for defense against *F. graminearum* infection. Our study highlights the critical role of rapid activation of defense pathways in the early response to *F. graminearum* in wheat scab resistance, which is required for activation and establishment of defense signaling cascades.

## Conclusions

To investigate the possible molecular mechanisms involved in wheat spikes defense against the *F. graminearum* infection, a phosphoproteomics analysis using antibodies against p-Tyr, p-Ser and p-Thr residues was performed to identify putative phosphoproteins. Finally, thirty-five phosphorylated signals were detected and protein identities of twenty-eight spots were determined. These proteins were mainly implicated in three functional groups, including defense/stress response, signal transduction, and metabolism. Bioinformatic analysis predicted that all the identified proteins contain at least one tyrosine, serine or/and threonine phosphorylation site. By further analysis of typical proteins from different groups, it is presumed that a significant change of the phosphorylation status of proteins for metabolism and regulatory pathways in resistant wheat line due to the *F. graminearum* infection exists, such as scavenging of ROS, the production of phytoalexin, the improvement of the SA content, the fortification of cell wall, and a wide range of other metabolic changes. Further cloning and functional analysis of these FHB-responsive putative phosphoproteins using genetic or other approaches will provide new insights into molecular mechanisms of wheat scab resistant.

Currently some techniques for the quantitative study of peptides, such as isobaric tags for relative and absolute quantitation (iTRAQ) and isotope-coded affinity tags (iCAT), are also useful for phosphoproteome analysis. Western blot analysis may combine other techniques to obtain complete patterns of the phosphoproteome. In addition, a major challenge in phosphoproteome analysis is the low abundances of many key phosphorylated regulatory proteins, which are difficult to detect and identify.

### *Author contribution statement*

LD and GY conceived and designed research. LD, RY and PL conducted experiments. GY and JC contributed new reagents or analytical tools. LD, YZ and JC analyzed data. LD wrote the manuscript. All authors read and approved the manuscript.

## Electronic supplementary material

Supplementary material 1 (DOC 31 kb)

## Abbreviations

FHB: *Fusarium* head blight
MALDI-TOF: Matrix-assisted laser desorption ionization time-of-flight
PVDF: Polyvinylidene fluoride
