# Supplementary material for: Identification of putative phosphoproteins in wheat spikes induced by Fusarium graminearum
Source: Planta. 2015 Dec 15;243:719–31. doi: 10.1007/s00425-015-2441-y (PMC4757628; doi:10.1007/s00425-015-2441-y)
Supplement: Supplementary file 1 — Supplementary material 1 (DOC 31 kb) [file 425_2015_2441_MOESM1_ESM.doc]

**Fig. S1**

**Fig. S1** The protein level for the control and 6 h after infection. Protein abundance was quantified using PDQuest software and plotted as the relative intensity. Data show a representative experiment from two independent experiments with similar results and three replicates each. The error bars indicate SD of three replicates.
